# Targeted inhibition of Hedgehog-GLI signaling by novel acylguanidine derivatives inhibits melanoma cell growth by inducing replication stress and mitotic catastrophe

**DOI:** 10.1038/s41419-017-0142-0

**Published:** 2018-02-02

**Authors:** Silvia Pietrobono, Roberta Santini, Sinforosa Gagliardi, Francesca Dapporto, David Colecchia, Mario Chiariello, Cosima Leone, Massimo Valoti, Fabrizio Manetti, Elena Petricci, Maurizio Taddei, Barbara Stecca

**Affiliations:** 1 Core Research Laboratory, Istituto Toscano Tumori, Florence, Italy; 2 Consiglio Nazionale delle Ricerche, Istituto di Fisiologia Clinica and Core Research Laboratory, Istituto Toscano Tumori, AOU Senese, Siena, Italy; 3 Department of Life Sciences, University of Siena, Siena, Italy; 4 Department of Biotechnology, Chemistry and Pharmacy, University of Siena, Siena, Italy; 5 Department of Oncology, Careggi University Hospital, Florence, Italy

## Abstract

Aberrant activation of the Hedgehog (HH) signaling is a critical driver in tumorigenesis. The Smoothened (SMO) receptor is one of the major upstream transducers of the HH pathway and a target for the development of anticancer agents. The SMO inhibitor Vismodegib (GDC-0449/Erivedge) has been approved for treatment of basal cell carcinoma. However, the emergence of resistance during Vismodegib treatment and the occurrence of numerous side effects limit its use. Our group has recently discovered and developed novel and potent SMO inhibitors based on acylguanidine or acylthiourea scaffolds. Here, we show that the two acylguanidine analogs, compound (**1**) and its novel fluoride derivative (**2**), strongly reduce growth and self-renewal of melanoma cells, inhibiting the level of the HH signaling target GLI1 in a dose-dependent manner. Both compounds induce apoptosis and DNA damage through the ATR/CHK1 axis. Mechanistically, they prevent G2 to M cell cycle transition, and induce signs of mitotic aberrations ultimately leading to mitotic catastrophe. In a melanoma xenograft mouse model, systemic treatment with **1** produced a remarkable inhibition of tumor growth without body weight loss in mice. Our data highlight a novel route for cell death induction by SMO inhibitors and support their use in therapeutic approaches for melanoma and, possibly, other types of cancer with active HH signaling.

## Introduction

Hedgehog (HH) signaling is a conserved pathway that plays a pivotal role during embryonic development, tissue homeostasis, and regeneration^1,2^. In vertebrates, canonical HH pathway activation is triggered by binding of secreted HH ligands to the 12-pass transmembrane receptor Patched (PTCH1) on nearby cells. The binding abolishes repression on the G protein-coupled receptor Smoothened (SMO), initiating an intracellular signaling cascade that regulates the formation of the zinc-finger transcription factors GLI2 and GLI3, which induce transcription of GLI1. Both GLI1 and GLI2 control the transcription of a number of context-dependent target genes that regulate cellular differentiation, proliferation, survival, and self-renewal.

Aberrant activation of the HH pathway has been reported to drive tumor progression in numerous cancers, including those of the skin, brain, lung, pancreas, stomach, and hematopoietic malignancies^3–5^. The development of small molecules targeting the HH signaling is a promising approach for the treatment of HH-dependent tumors. Starting from the natural compound Cyclopamine, an alkaloid isolated from *Veratrum californicum* that attenuates HH signaling by antagonizing SMO^6,7^, several SMO antagonists have been identified so far^8,9^. Among them, Vismodegib (GDC-0449/Erivedge) and Sonidegib (LDE-225/Odomzo) have been approved by FDA for treatment of locally advanced or metastatic basal cell carcinoma. However, despite an initial clinical response, the use of SMO inhibitors has been associated with the acquisition of tumor drug resistance as a result of structural mutations in SMO^10–12^. In addition, Vismodegib and Sonidegib can trigger a number of side effects, including constipation, diarrhea, hair loss, and fatigue. Several clinical trials with SMO antagonists led to negative results due to low selectivity on cancer stem cells (CSCs), poor pharmacokinetic properties, and the occurrence of mechanisms of non-canonical HH pathway activation downstream of SMO^13,14^. Resistance to SMO inhibitors can be mediated by amplification of the HH target genes *GLI2* and *CyclinD1* (ref. 15) or upregulation of GLI by non-canonical HH pathway^16^. Therefore, there is a need for new SMO antagonists able to effectively inhibit tumor growth and CSC self-renewal, while avoiding drug resistance mechanisms.

Our group has recently developed a series of novel SMO inhibitors based on acylguanidine or acylthiourea scaffolds^17^. In particular, compound **1** (MRT-92) was shown to uniquely bind to the entire transmembrane cavity of SMO and to be insensitive to the human D473H^18^, a key mutation that renders SMO resistant to Vismodegib^10^ or Sonidegib^16^. Compound **1** is among the most potent SMO antagonists known so far, being 10-fold more potent than Vismodegib or Sonidegib in inhibiting rat cerebellar granule cell proliferation^18^. However, the biological effects of these acylguanidine and acylthiourea derivatives in human melanoma cells remain to be determined. Here we show that **1** inhibits GLI1 expression and reduces melanoma cell growth *in vitro* and *in vivo*, by inducing DNA damage and G2/M cell cycle arrest.

## Results

### Inhibition of endogenous HH signaling by **1** and **2**

**Fig. 1:**
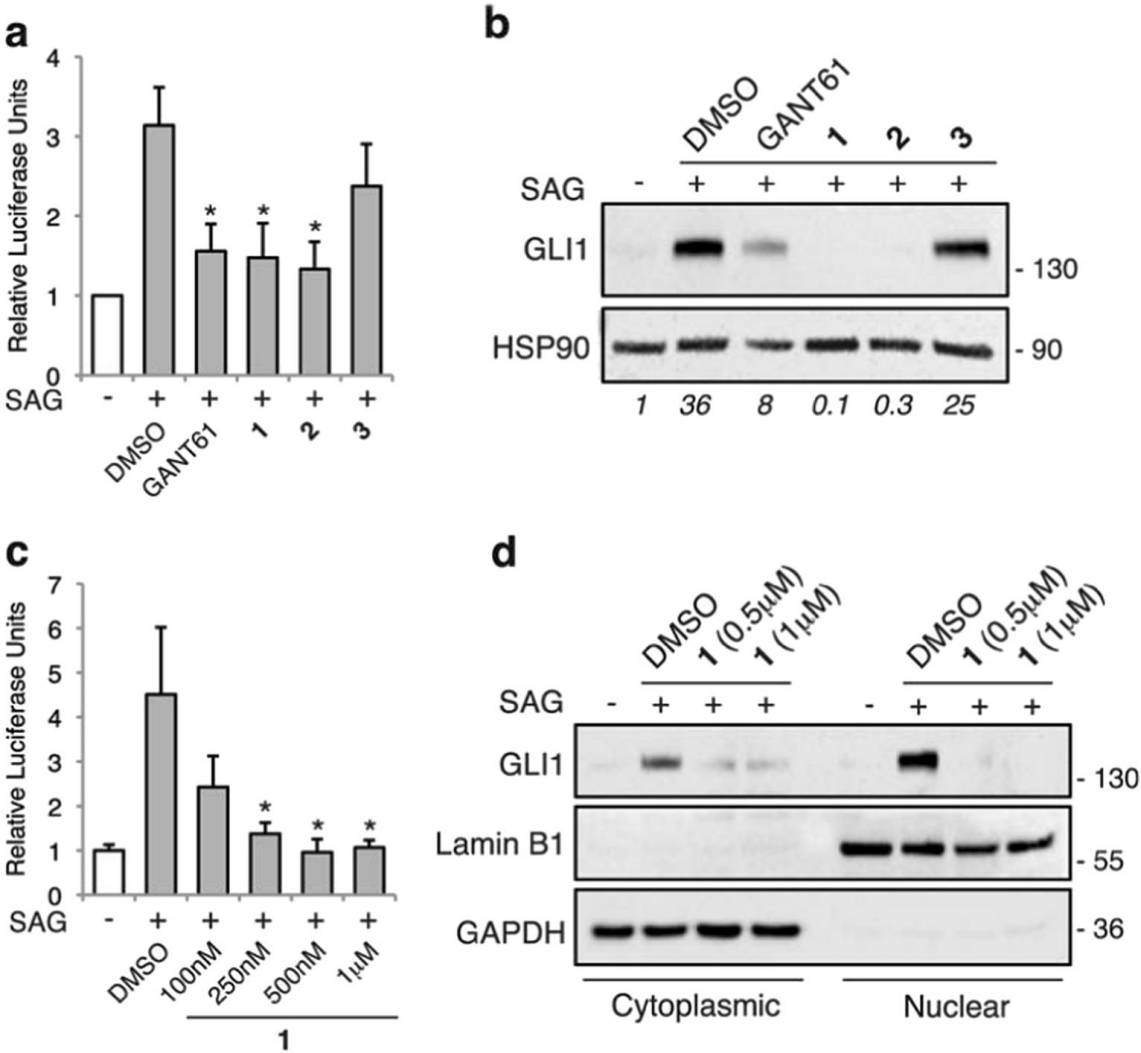
Effect of compounds **1**, **2**, and **3** on the endogenous HH pathway **(a)** Quantification of GLI-dependent luciferase reporter assay in HH-responsive NIH3T3 cells treated with 100 nM SAG and GANT61 (5 μM), **1**, **2**, or **3** (1 μM). Cells were treated with SAG for 48 h and with GANT61 or compounds for 24 h. Relative luciferase units were GLI-dependent reporter firefly/renilla control ratios, with untreated cells equated to 1. **(b)** Western blot (WB) analysis of endogenous GLI1 protein in NIH3T3 cells treated with 100 nM SAG and GANT61 (5 μM), and **1**, **2**, or **3** (1 μM) for 48 h. HSP90 was used as loading control. Quantification of GLI1 protein, expressed as relative ratio of GLI1/HSP90, is shown in italic. **(c)** Quantification of GLI-dependent luciferase reporter assay in HH-responsive NIH3T3 cells treated with 100 nM SAG and increasing doses of **1** for 48 h. Relative luciferase units were GLI-dependent reporter firefly/renilla control ratios, with untreated cells equated to 1. **(d**) WB analysis of cytoplasmic and nuclear endogenous GLI1 protein in NIH3T3 cells treated with 100 nM SAG and **1** at the indicated doses for 48 h. After treatment, cell fractionation was performed and lysates were subjected to WB with anti-GAPDH (control for cytoplasmic proteins) and anti-Lamin B1 (control for nuclear proteins). Data are shown as mean ± SD of at least three independent experiments. ^*^p < 0.05 compared with DMSO control.

The inhibitory properties of **1**, **2**, and **3** on the transcriptional activity of the HH pathway were investigated using the HH-competent murine NIH3T3 cells transiently transfected with a GLI-binding site firefly luciferase reporter and treated with the SMO agonist SAG^19^. Compounds **1** and **2** reduced the transcriptional activity of the endogenous HH pathway by 50%, to a level comparable to the GLI inhibitor GANT61 (ref. 20) whereas **3** did not affect it (Fig. 1a). Consistently, western blot analysis in NIH3T3 cells showed that only **1** and **2** reduced the expression of endogenous Gli1, the best read-out of an active HH pathway^21^ (Fig. 1b). Compound **1** showed a dose-response ability to inhibit the transcriptional activity of the endogenous HH pathway (Fig. 1c). Synthesis of compounds **1**, **2,** and **3** is reported in Supplementary Figure S1.

GLI1 shuttles between the cytoplasm and the nucleus, where it induces the expression of target genes^22^. Therefore, we tested whether **1** affects the intracellular trafficking of Gli1. As expected, SAG treatment induced the expression of Gli1 protein in the nucleus, and, to a lesser extent, in the cytoplasm. Treatment with **1** resulted in a strong Gli1 inhibition in both the nucleus and the cytoplasm (Fig. 1d), suggesting that **1** does not affect the intracellular trafficking of Gli1, but it rather inhibits its expression. To further confirm the specificity of action of **1** for the HH signaling, no substantial inhibition of any of the 46 analyzed kinases was detected upon treatment with **1** (Supplementary Figure S2).

Altogether these data indicate that **1** and **2** show a very strong inhibitory activity against the HH pathway, whereas **3** shows no ability to suppress it. The guanidine moiety of **1** and **2** appears to be crucial for the HH inhibitory activity, because its replacement by a thiourea lead to inactive compound **3** (Table 1).

**Table 1:** Summary of IC_50_ values on melanoma cell viability and inhibition of endogenous Gli1 protein

Compound	Chemical structure	IC_50_ (nM) on cell viability^*^			Gli1 protein inhibition, %^**^
		A375	SSM2c	MeWo	
**1** (MRT-92)		299 ± 0.05	393 ± 0.1	614 ± 0.05	99 ± 0.5
**2**		368 ± 0.07	391 ± 0.04	608 ± 0.07	99 ± 0.8
**3** (MRT-95)		nd	nd	nd	31 ± 5

^*^IC_50_ values were calculated using GraphPad; nd: IC_50_ cannot be determined by GraphPad. Data represent mean ± SEM

^**^Gli1 protein levels were determined in NIH3T3 cells stimulated with SAG 100 nM and treated with 1 μM of each compound for 48 h. Data represent mean ± SD

### Compounds **1** and **2** inhibit melanoma cell viability

Compound **1** has been shown to inhibit proliferation of rat cerebellar granule cells and of murine Ptch1^+/−^ medulloblastoma cells at nanomolar concentrations^18^. However, it is unknown whether **1** or **2** have anti-proliferative activity in human cancer cells.

**Fig. 2:**
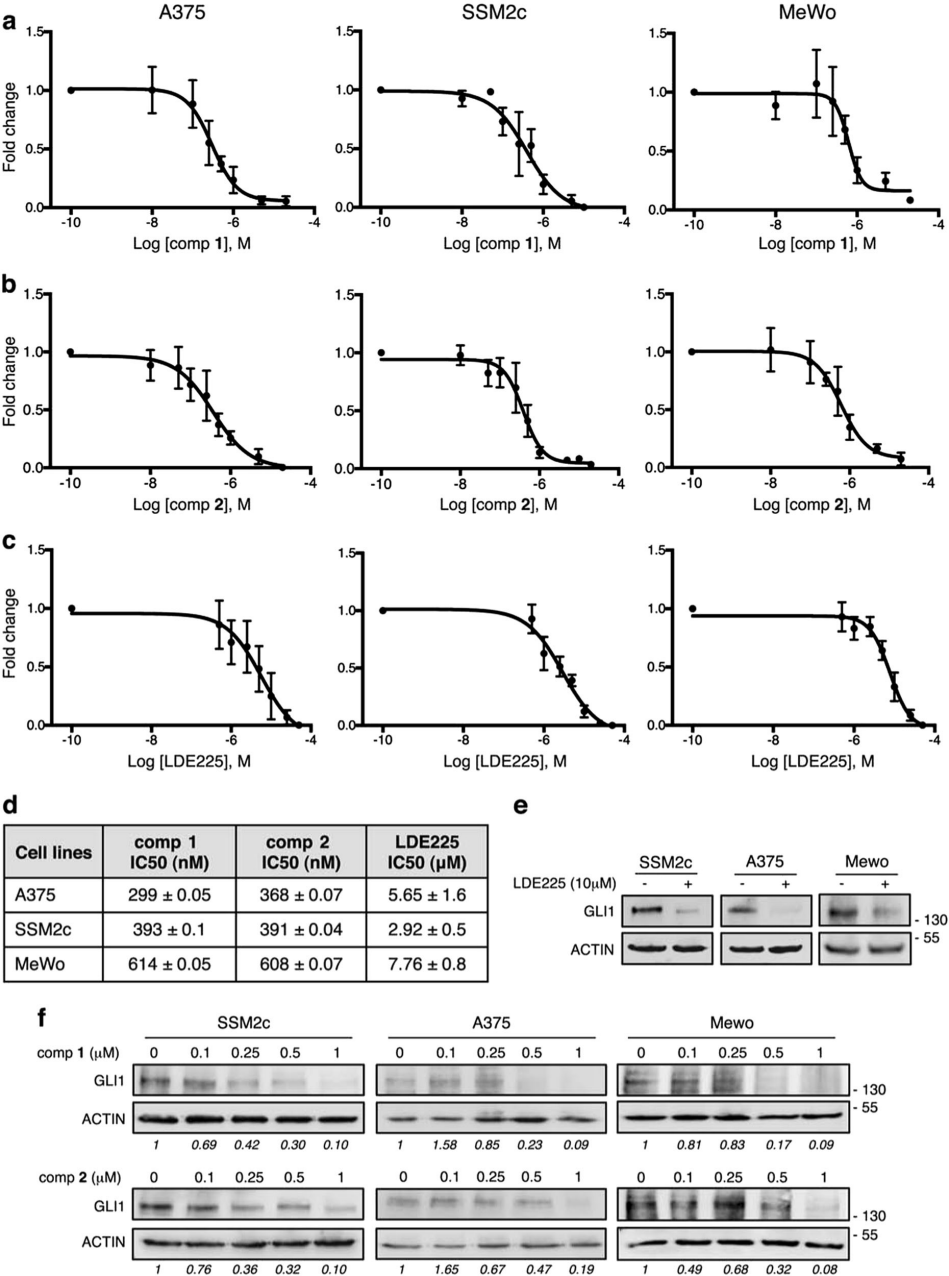
Compounds **1** and **2** inhibit melanoma cell growth in a dose-dependent manner **(a**-**c)** Dose-response curves of **1 (a)**, **2 (b**), and LDE-225 **(c)** in A375, SSM2c, and MeWo melanoma cells treated with vehicle (DMSO) or increasing doses of each drug for 72 h. Curves were obtained using GraphPad. **(d**) Table reports IC_50_ values for each cell line. Data represent mean ± SEM of at least three independent experiments. **(e)** Western blot analysis of GLI1 in SSM2c, A375, and MeWo cells treated with DMSO or LDE-225 (10 μM) for 48 h. **(f)** Western blot analysis of GLI1 in SSM2c, A375, and MeWo cells treated with DMSO (0) or increasing doses of **1** or **2** for 48 h. ACTIN was used as loading control. Quantification of GLI1 protein, expressed as relative ratio of GLI1/ACTIN, is shown in Italic.

Along with others, we previously showed that inhibition of SMO reduces growth of human melanoma cell lines *in vitro* and *in vivo*^23–25^. Thus, we tested the ability of **1** and **2** to suppress proliferation of human melanoma cells compared with the SMO antagonist LDE-225. Treatment of A375, SSM2c, and MeWo melanoma cells with **1** and **2** showed a dose-dependent reduction of cell viability in all three cell types, with IC_50_ concentrations ranging from 299 to 614 nM for **1** and from 368 to 608 nM for **2** (Figs. 2a–d). On the other hand, LDE-225 showed IC_50_ in the μM range (Figs. 2d, e) and acted mainly through induction of apoptosis (Supplementary Figure S3), as previously reported^24^. Western blot analysis showed that both **1** and **2** reduced the expression of endogenous GLI1 protein in a dose-dependent manner in all three melanoma cell lines (Fig. 2f). These data indicate that both compounds are able to restrain melanoma cell growth *in vitro* by inhibiting the expression of GLI1.

### Compounds **1** and **2** induce DNA damage and apoptosis in melanoma cells

**Fig. 3:**
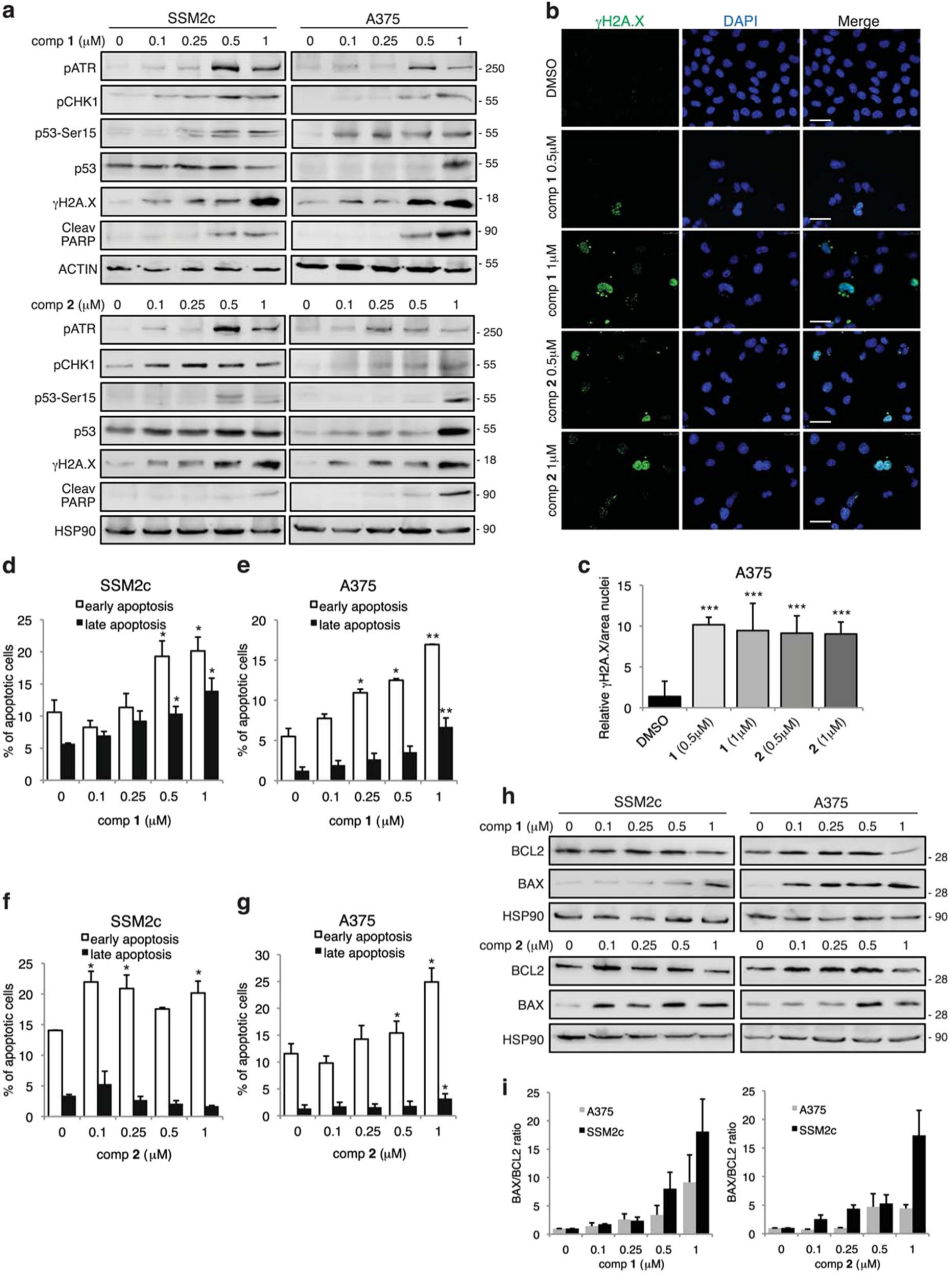
Effects of compounds **1** and **2** on apoptosis and DNA damage **(a)** Western blot analysis of DNA damage markers in SSM2c and A375 cells treated with DMSO (0) or increasing doses of **1** or **2** for 48 h. ACTIN or HSP90 were used as loading controls. **(b)** Confocal images of γH2A.X in A375 melanoma cells treated with compounds **1** or **2** for 48 h. Scale bar = 40 μm. **(c)** Quantification of γH2A.X in A375 cells as shown in **b**. (**d-g)** Evaluation of cell death by Annexin V/7-AAD staining in A375 and SSM2c cells treated with DMSO or increasing doses of **1 (d-e)** or **2 (f–g)**. **(h)** Western blot analysis of apoptotic markers in SSM2c and A375 cells treated with DMSO (0) or increasing doses of **1** or **2** for 48 h. HSP90 was used as loading control. **(i)** Densitometric quantification of BAX/BCL2 ratio in SSM2c and A375 cells treated as indicated in **h**. Data are shown as mean ± SD (**c)** and mean ± SEM **(d-g**, **i)** of at least three independent experiments. ^*^p < 0.05; ^**^p < 0.01; ^***^p < 0.001 compared with DMSO control.

The efficacy of many anticancer drugs relies on their ability to induce damage to cellular DNA and subsequent apoptosis^26^. To determine whether **1** or **2** induce DNA damage, we examined the activities of ataxia–telangiectasia mutated (ATM) and ataxia–telangiectasia and Rad3 related protein (ATR), two kinases that are critical for the DNA damage response through the activation of cell cycle checkpoints. Western blot analysis showed that both compounds increased DNA damage in melanoma cells by inducing phosphorylation of ATR and consequently activation of the downstream target pCHK1. Consistently, p53 was phosphorylated in the N-terminal activation domain at Ser15 (ref. 27) (Fig. 3a). In contrast, phosphorylation of ATM and, hence, activation of pCHK2 remained undetectable after treatment with either drugs (Supplementary Figure S4). To further characterize the DNA damage response, the expression of poly ADP-ribose polymerase-1 (PARP-1), a DNA damage sensor activated by DNA lesions^28^, and γH2AX, which can be induced by ATR in response to single-stranded DNA breaks and during replication stress^29^, were determined by western blot. Treatment of SSM2c, A375, and MeWo cells with both compounds induced cleavage of PARP-1 and a dose-dependent increase of phosphorylated γH2AX (Fig. 3a; Supplementary Figure S5a). Strong accumulation of γH2AX foci was also confirmed by confocal microscopy (Figs. 3b, c). These data suggest that both **1** and **2** induce DNA damage in melanoma cells through the ATR/CHK1 axis.

To determine whether **1** or **2** affected apoptosis, we performed analysis of Annexin V/7-amino-actinomycin D (7-AAD) labeling. Compound **1** led to a dose-dependent increase of both early and late apoptosis in A375 and SSM2c cells already after 48 h (Figs. 3d, e). Conversely, **2** was less efficient in inducing apoptosis, increasing only early apoptosis in SSM2c cells and both early and late apoptosis at the highest doses (0.5 and 1 μM) in A375 cells (Figs. 3f, g). Similarly, **1** was more effective than **2** in inducing apoptosis in MeWo cells (Supplementary Figure S5b and c). Induction of apoptosis was confirmed at the molecular level by increased BAX/BCL2 ratio (Figs. 3h, i; Supplementary Figure S5d), an indicator of mitochondrial apoptosis.^30^ These data indicate that **1** and, to a lesser extent, **2** induce apoptosis in melanoma cells.

### Compound **1** induces G2/M cell cycle arrest culminating in a process of mitotic catastrophe

**Fig. 4:**
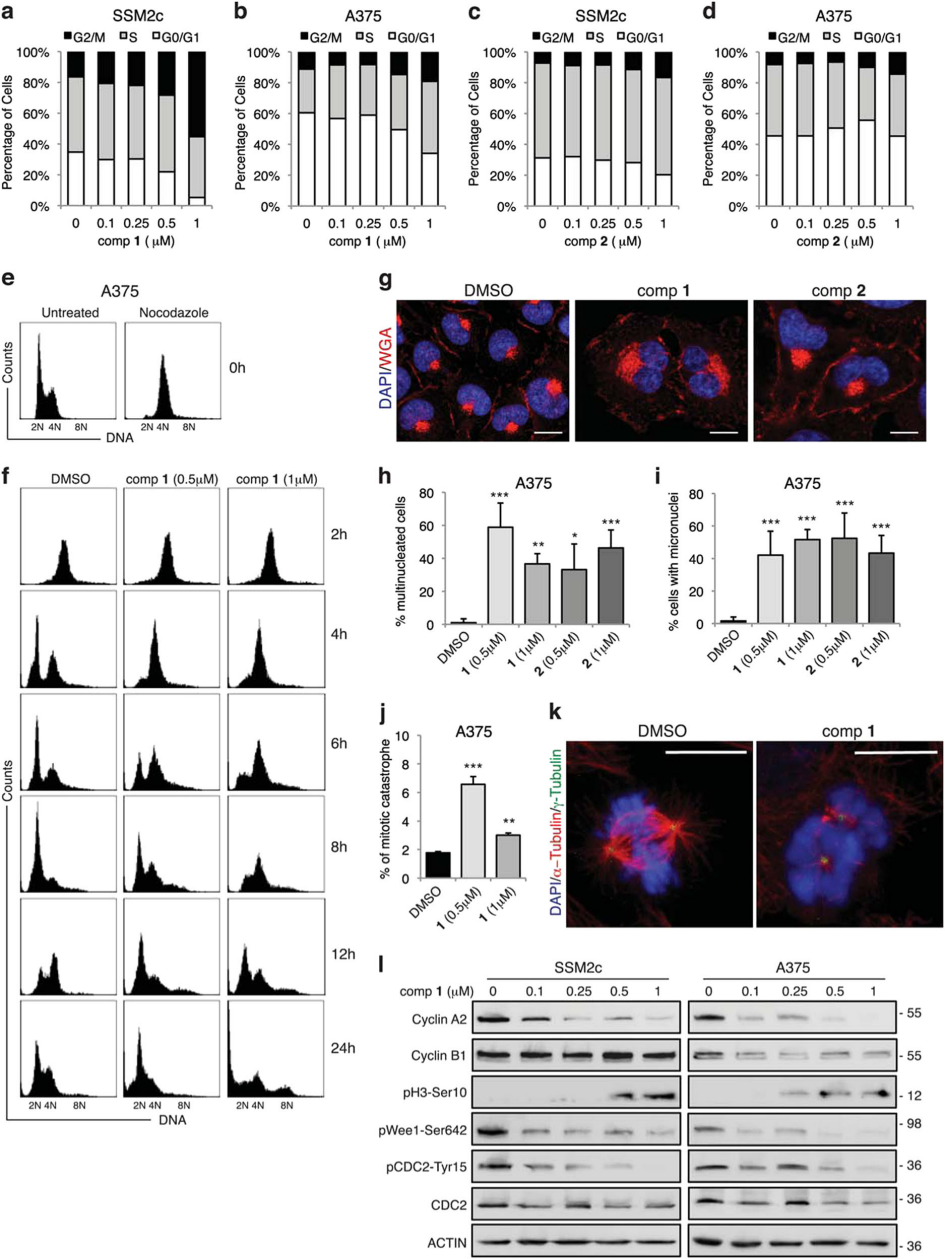
Compound **1** induces G2/M cell cycle arrest in melanoma cells **(a-d)** Cell cycle analysis in A375 and SSM2c cells treated with DMSO (0) or increasing doses of **1** or **2**. **(e)** Effect of nocodazole treatment (600 nM) for 16 h in A375 cells. **(f**) Cells were treated as indicated after release from nocodazole block and cell cycle distribution was determined by flow cytometric analysis of propidium iodide-stained cells collected at the indicated time points (right). Note that treatment with **1** prevents cell cycle progression after the released from nocodazole block. **(g)** Representative confocal microscopy images showing multinucleated A375 melanoma cells following treatment with DMSO and compounds **1** or **2** (1 μM for 48 h). Cells were incubated with WGA and nuclei were stained with DAPI. Scale bar = 10 μm. **(h)** Percentage of multinucleated A375 cells after treatment with DMSO, **1** or **2** for 48 h. (**i**) Percentage of A375 cells with micronuclei after treatment with DMSO, **1** or **2** for 48 h. **(j)** Percentage of A375 with mitotic catastrophe after treatment with **1** for 72 h. Cells were stained with α-tubulin (red), γ-tubulin (green), and DAPI (blue) to evaluate mitotic progression. Over 150 mitotic cells were observed and scored as normal or undergoing mitotic catastrophe. **(k)** Representative confocal microscopy images of A375 cells counted as normal mitosis (DMSO) or mitotic catastrophe after treatment with compound **1**. Cells were stained with α-tubulin, γ-tubulin, and DAPI. Scale bar = 10 μm. **(l**) Western blot analysis of cell cycle markers in SSM2c and A375 cells treated with DMSO (0) or increasing doses of compound **1** for 24 h. ACTIN was used as loading controls. Data are shown as mean ± SD **(h, i**) or mean ± SEM (**j)** of at least three independent experiments. ^*^p < 0.05; ^**^p < 0.01; ^***^p < 0.001 compared with DMSO control.

As the decrease in melanoma cell viability caused by **1** and **2** could be due to a decrease in cell proliferation, augmented cell death, or both, we examined the mechanism of cell growth inhibition. First, we measured how **1** and **2** impact on cell cycle by carrying out propidium iodide staining of SSM2c, A375, and MeWo cells treated with increasing doses of either compounds. Treatment with **1** and, to a lesser extent, **2** induced accumulation of SSM2c, MeWo, and A375 cells in the G2/M phase of the cell cycle with a concomitant decrease in the G0/G1 population compared with the control (Figs. 4a–d; Supplementary Figure S5e-f). To further investigate the mechanisms underlying the observed G2/M arrest, melanoma cells were synchronized in prometaphase with the microtubule-interfering agent nocodazole, and then released in absence or presence of either **1** or **2**. Fluorescence-activated cell sorting (FACS) analysis confirmed that A375 and SSM2c cells were arrested in mitosis after nocodazole blockade, as shown by the 4N DNA content of cells immediately following the block (Fig. 4e; Supplementary Figure S6a-b) (0 h release). After removal of the spindle poison, dimethyl sulfoxide (DMSO)-treated cells rapidly re-entered the cell cycle, and the majority of A375 and SSM2c cells were in G1 phase with 2N DNA content at 8 and 6 h, respectively. In contrast, the majority of A375 and SSM2c cells treated with **1** were still at G2/M phase, as indicated by the large fraction of cells with 4N DNA content at 8 h (SSM2c) and at 6 h (A375) (Fig. 4f; Supplementary Figure S6c). This delay in the production of G1 cells could be due to their inability to initiate anaphase or to exit mitosis. Prolonged observation of treated cells indicated that cells with a 4N DNA content failed to complete mitosis, as shown by the appearance of a 8N DNA content already after 8 h, thus suggesting mitotic catastrophe. Notably, higher doses of **1** induced increase of the subG0 fraction in both SSM2c and A375 cells after 24 h, which is compatible with the activation of the “mitotic death” program of mitotic catastrophe (Fig. 4f; Supplementary Figure S6c).

Failing mitoses are often associated with gross nuclear alterations, such as multinucleation and micronucleation, which constitute the most prominent morphological traits of mitotic catastrophe^31^. Indeed, confocal microscopy examination revealed that **1** induced the formation of multinucleated cells (Figs. 4g, h), similarly to what observed by cell cycle analysis (Fig. 4f), with a very high percentage of cells containing several micronuclei (Fig. 4i). Compound **2** was less effective in delaying mitotic exit, since 12 h (for SSM2c) and 8 h (for A375) after release most of the cells progressed into the G1 phase (Supplementary Figure S7) but, nonetheless, showed high percentage of multinucleated and micronucleated cells (Figs. 4g–i). Confocal microscopy showed disruption of the spindle apparatus upon treatment with **1**, confirming the induction of mitotic catastrophe (Figs. 4j, k).

At the molecular level, treatment with **1** led to a dose-dependent reduction of cyclin A2, whose inhibition occurs upon entry into mitosis^32,33^, and induced phosphorylation of histone H3 (pH3-Ser10), which is associated with chromosome condensation and mitotic entry^34^ (Fig. 4l). Of note, both compounds decreased the activity of Wee1 (pWee1-Ser642), which is involved in G2/M cell cycle checkpoint arrest to allow DNA repair before mitotic entry^35,36^. As a consequence, CDC2 is activated, as shown by decreased phosphorylation of the Tyrosine 15 (pCDC2-Tyr15) (Fig. 4l; Supplementary Figure S5g), suggesting that **1** and **2** induced a forced passage through the G2 checkpoint despite large DNA damage, which may lead to apoptosis in response to replication stress (Fig. 3; Supplementary Figure S5)^37^. These data suggest that **1** and, to a lesser extent, **2** delay exit of melanoma cells from mitosis by inducing signs of mitotic catastrophe.

As treatment of melanoma cells with compounds induces activation of p53 (Fig. 3a), we investigated whether p53 could mediate the effects of these compounds by silencing it. Cell cycle analysis showed that **1** and **2** induced G2/M arrest in A375 and SSM2c cells transduced with both LV-c or LV-shp53, whereas late apoptosis occurred only in presence of p53 (Supplementary Figure S8). Nocodazole treatment revealed that p53-depleted cells re-entered cell cycle earlier than cells expressing p53, but accumulated in G2/M at later time points. Consistently, **1** prevented inactivation of pWee1 and induced activation of pCDC2 in absence of p53 (Supplementary Figure S9). Taken together, these results suggest that functional p53, although not required for compound **1**-induced G2/M cell cycle arrest, might play a role in mediating mitotic catastrophe.

### Compounds **1** and **2** suppress self-renewal of melanoma stem-like cells

**Fig. 5:**
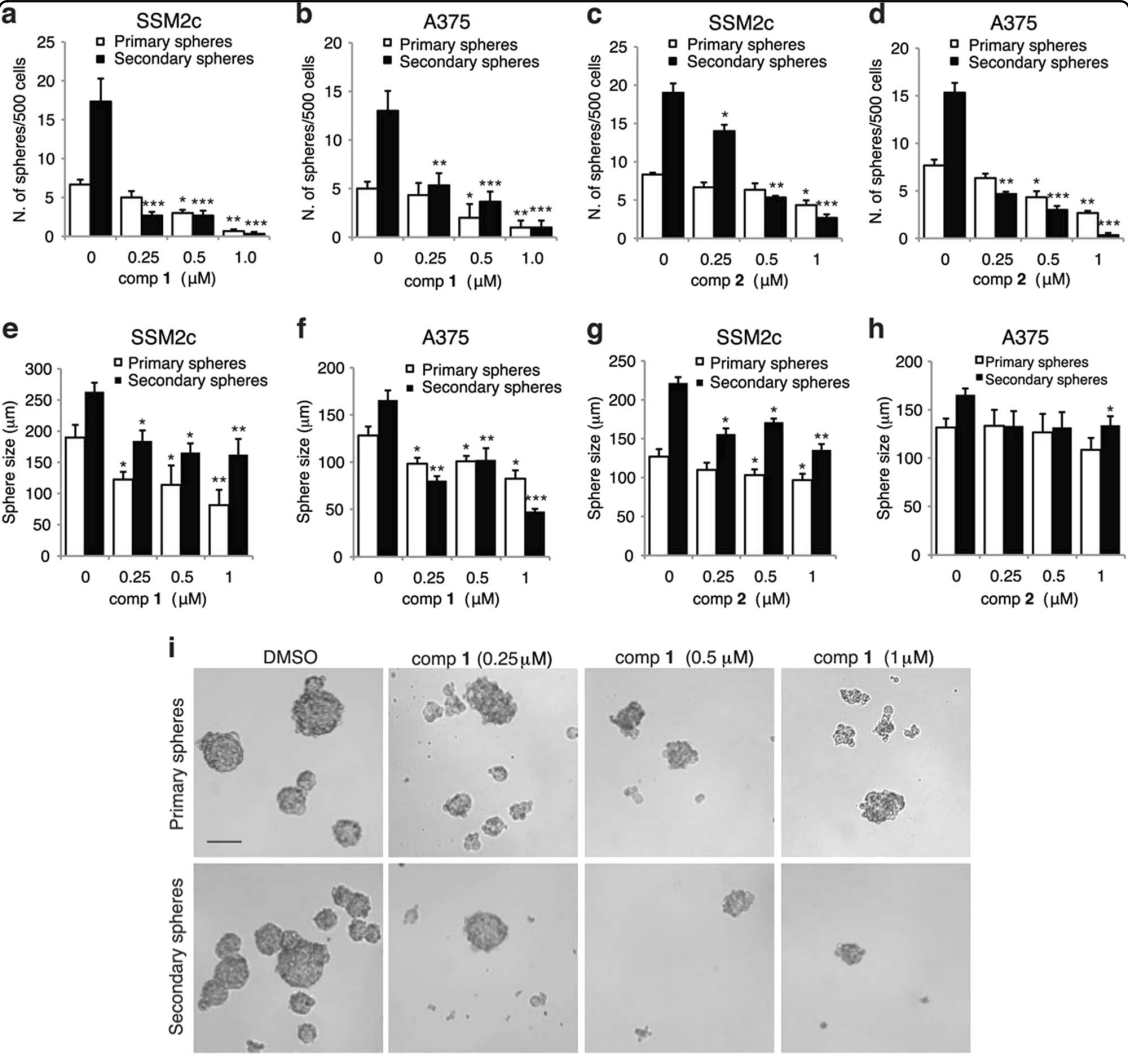
Compounds **1** and **2** inhibit self-renewal of melanoma stem-like cells **(a-d)** Effects of **1 (a, b)** and **2 (c, d**) on primary and secondary spheres from SSM2c **(a, c)** and A375 (**b, d**) melanoma cells. **(e-h)** Spheres size, as indicated in **a-d**. Melanoma spheres were treated at the indicated doses for 96 h during primary sphere formation and left untreated to form secondary spheres. In all, 500 single cells were plated and the number of spheres larger than 50 μm was counted after 1 week. Data represent mean ± SEM of three independent experiments. (**i**) Representative phase contrast images of primary and secondary SSM2c spheres as indicated in **a**. Scale bar = 100 μm. ^*^p < 0.05; ^**^p < 0.01; ^***^p < 0.001 compared with DMSO control (0)

HH signaling plays a pivotal role in the maintenance and self-renewal of stem-like cells in several types of cancer^38^. The presence of these cells in the tumor mass is a major cause of resistance and favors tumor relapse. However, to date SMO inhibitors have demonstrated low selectively against stem-like cells^39^. Therefore, we tested whether **1** and **2** affect clonogenic self-renewal ability of non-adherent melanoma spheres, which are enriched in CSCs^40,41^. Treatment of A375 and SSM2c cells with increasing doses (0.1, 0.25, 0.5, 1 μM) of either compounds negatively affected their ability to form primary spheres and led to a progressive and almost complete loss of their ability to self-renew and form secondary spheres from single-cell suspension (Figs. 5a–d). Primary and secondary spheres treated with both compounds were also reduced in size (Figs. 5e–i), thus suggesting an effect on progenitors or more differentiated cells composing the sphere. Altogether, these data indicate that both **1** and **2** drastically reduce the ability of melanoma spheres to self-renew *in vitro*, suggesting that these compounds have high selectively against melanoma CSCs.

### Silencing of SMO abolishes the effect of **1** and **2** on melanoma cell viability

**Fig. 6:**
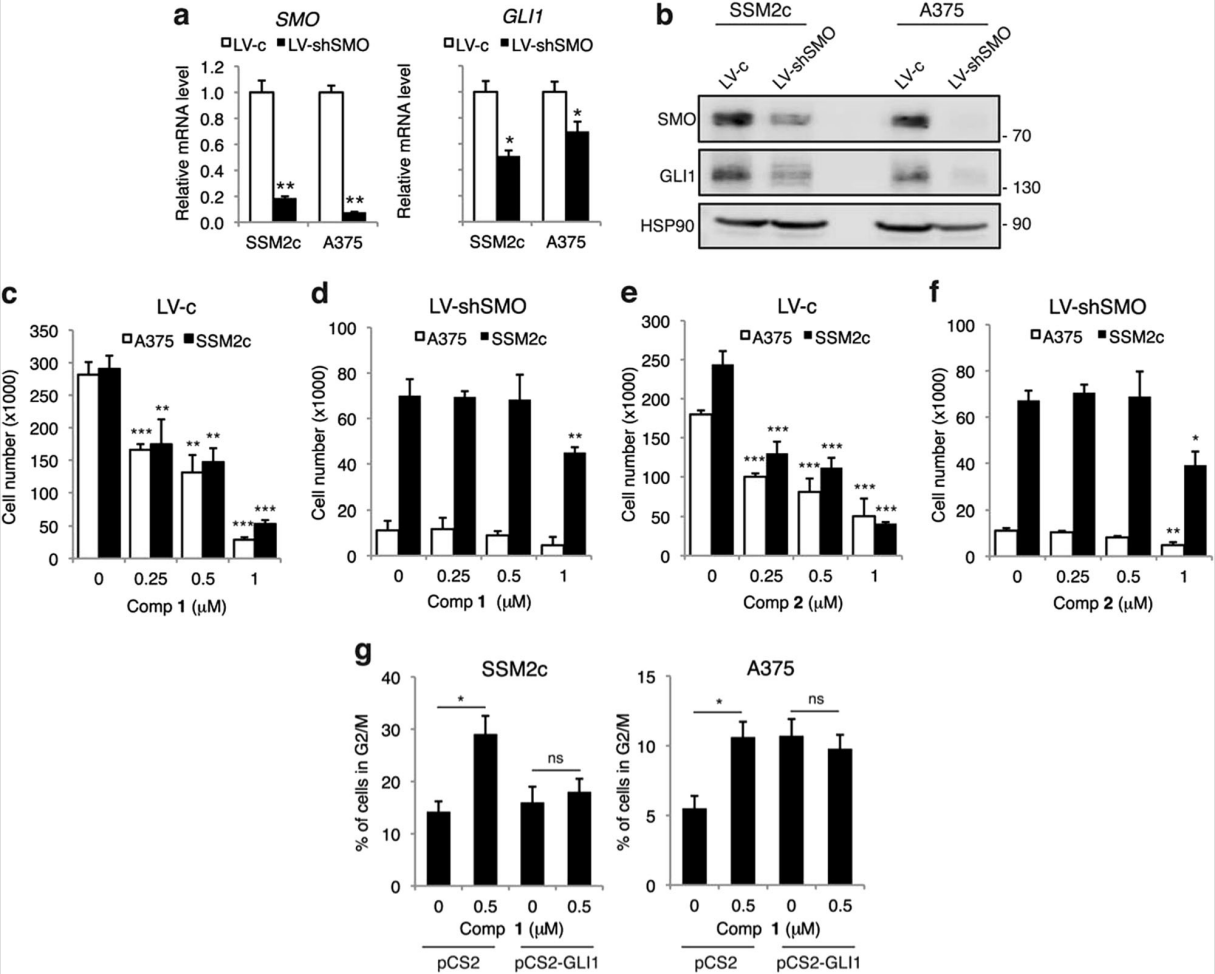
Silencing of SMO abolishes the effect of **1** and **2** on melanoma cell viability **(a)** Quantitative real-time PCR (qPCR) analysis of *SMO* and *GLI1* mRNA in SSM2c and A375 cells treated as indicated. The *y* axis represents expression ratio of gene/(GAPDH and β-ACTIN average), with the level of control equated to 1. **(b)** Western blot analysis of SMO and GLI1 in A375 and SSM2c melanoma cells transduced with LV-c or LV-shSMO. HSP90 was used as loading control. **(c-f**) Effect of **1 (c-d)** and **2** **(e**-**f**) on viability of A375 and SSM2c melanoma cells transduced with LV-c or LV-shSMO. Cells were treated with DMSO (0), **1** or **2** at the indicated doses for 72 h. (**g)** Percentage of SSM2c and A375 cells in G2/M phase upon transient transfection of pCS2 or pCS2-GLI1 and treatment with DMSO (0) or **1**. Data are shown as mean ± SD of at least three independent experiments. ^*^p < 0.05; ^**^p < 0.01; ^***^p < 0.001 compared with DMSO control

To confirm the specificity of **1** and **2** for SMO, we silenced it in melanoma cells using a short hairpin RNA specific for *SMO*^40^ and we treated them with increasing concentrations of either compounds. Silencing of *SMO* drastically reduced the expression of SMO and GLI1 mRNA and protein in both A375 and SSM2c cells (Figs. 6a, b), as expected. Silencing of *SMO* strongly inhibited proliferation of melanoma cells compared with control LV-c in both cell types (Figs. 6d *vs* c and f *vs* e), as previously shown^40^. Noteworthy, treatment with increasing doses of either compounds reduced viability of melanoma cells transduced with LV-c control (Figs. 6c, e). On the contrary, treatment of SMO-depleted melanoma cells (LV-shSMO) with both compounds showed a minor effect only at 1 μM in both cell types (Figs. 6d, f). To further confirm that these compounds act through the inhibition of the HH signaling to exert their anti-proliferative and mitotic catastrophe effects, we transiently overexpressed GLI1 in presence of compound **1**. Cell cycle analysis showed that GLI1 rescued the effect of **1** on G2/M cell cycle arrest (Fig. 6g). Ectopic GLI1 prevented ATR activation and p53 induction (Supplementary Figure 9d), consistently with the previously described negative autoregulatory loop between p53 and GLI1^42–44^. This finding suggests that the p53-mediated mitotic catastrophe likely occurs downstream of GLI1.

### Compound **1** inhibits growth of human melanoma xenografts

**Fig. 7:**
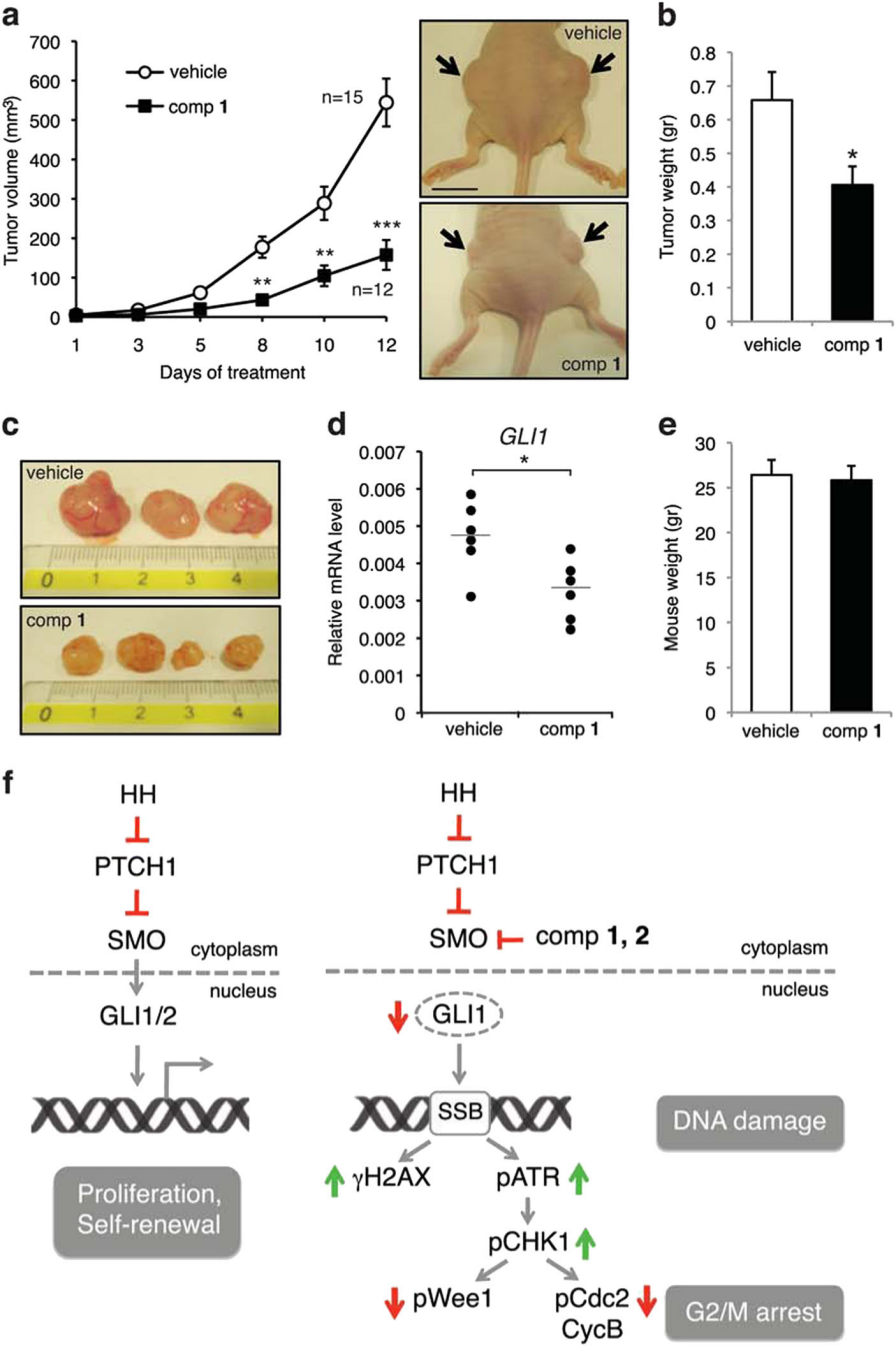
Inhibition of melanoma xenograft growth ***in vivo*** by **1** and mechanism of action **(a)** *In vivo* tumor growth of A375 melanoma cells subcutaneously injected into athymic nude mice. Animals were treated at tumor appearance with vehicle or compound **1** (15 mg/kg). After 12 days of treatment, mice were sacrificed. Number of tumors for each group is indicated. Representative images of A375 xenografts, as indicated. Scale bar = 10 mm. **(b)** Tumor weight in mice treated with vehicle or compound **1**. **(c)** Representative tumor size in mice treated with vehicle or compound **1**. **(d)** qPCR of *GLI1* mRNA in melanoma xenografts in mice treated with vehicle or compound **1** (six tumors were analyzed for each group). The *y* axis represents expression ratio of gene/(GAPDH and TBP average). **(e)** Mice body weight at the end of the treatment. Data shown are mean ± SEM **(a, b)** or mean ± SD **(e).** ^*^p < 0.05; ^**^p < 0.01; ^***^p < 0.001 compared with vehicle control. **(f**) Schematic representation of the mechanism underlying the inhibition of HH pathway that results in G2/M arrest. Targeting SMO with **1** and **2** blocks GLI1 function and induces DNA damage with formation of γH2AX foci, activation of the ATR-CHK1 axis and sign of mitotic catastrophe, as observed by increased chromatin condensation (pH3-Ser10), multinucleation, presence of micronuclei, and aberrant mitotic spindle (see Fig. 5 for details)

To investigate the inhibitory effect of **1** on tumor growth *in vivo*, A375 melanoma cells were subcutaneously injected into athymic nude mice and when tumors were palpable mice were randomized and treated twice a day with intraperitoneal (i.p.) injections of **1** (15 mg/kg) or vehicle alone. Treatment with **1** produced a significant reduction in tumor growth compared with vehicle (Figs. 7a–c), consistently with decreased expression of *GLI1* mRNA (Fig. 7d). No significant changes in body weight of the animals were observed, indicating good tolerance of the doses of **1** (Fig. 7e). These results further confirm **1** to be effective in the inhibition of the HH signaling and a potent anticancer agent *in vivo* with negligible systemic toxicity.

## Discussion

The HH signaling pathway plays a critical role in the initiation and progression of several types of cancer. SMO, which is one of the major components of the HH pathway, transduces the signal in receiving cells, triggering an intracellular cascade that ultimately leads to the activation of the GLI transcription factors^45^. SMO is also the target of several small-molecule inhibitors for anticancer therapies^8,9^. The clinical development of SMO antagonists has been undermined by several factors, including the emergence of drug resistance, severe side effects, poor pharmacokinetic properties, and low selectivity on CSCs^13,14^.

Recently, novel acylguanidine and acylthiourea derivatives targeting SMO have been discovered^17^. However, the biological effects of these drugs were not addressed, nor the expression of the downstream mediator GLI1 was investigated. In this study, we report the synthesis of the acylthiourea **3** (MRT-95), the acylguanidine **1** (MRT-92) and its fluoride derivative **2**. We provide evidence that **1** and **2** are able to reduce viability of melanoma cells, with nanomolar IC_50_ (Table 1), and to inhibit self-renewal of melanoma stem-like cells. In addition, **1** drastically reduces growth of human melanoma xenografts. Compounds **1** and **2** appear to be specific toward the HH pathway, as both are able to drastically inhibit Gli1 protein expression in murine NIH3T3 cells and in human melanoma cells without significant effect on a panel of 46 kinases. On the contrary, the substitution of the guanidine moiety with a thiourea (**3**) abolishes the inhibitory activity on the HH signaling, thus suggesting that the guanidine moiety present in **1** and **2** is critical for their activity. Metabolic stability studies of **1** showed that the principal metabolite is the *O*-demetylated compound either in rat liver microsomes (RLM) and human liver microsomes (HLM). The formation of this metabolite, however, cannot affect the pharmacological properties of **1** but can contribute to modify its pharmacokinetic properties, with the formation of more hydrophilic derivatives.

The clinical development of SMO antagonists has proved disappointing due to the low selectively against CSCs. Formation of clonogenic non-adherent melanoma spheres in serum-free media is a functional assay that has been used to investigate self-renewal ability of cancer cells with stem-like properties^41,46,47^. Our data demonstrate that **1** and **2** reduce the putative CSC population in both A375 and SSM2c melanoma cells already at 250 nM, paralleling results obtained with genetic silencing of SMO, GLI1 or treatment with GANT61 (ref. 40) These data suggest that both compounds have good selectivity against melanoma CSCs, making them promising candidates for further pre-clinical and clinical studies in melanoma and other types of cancer.

Our results revealed that treatment with **1** or **2** induces a replication stress that leads to the activation of the ATR/CHK1 DNA damage signaling cascade. In mammalian cells, there are two parallel pathways that respond to stress-induced DNA damage: ATM-CHK2, mostly driven by double-strand breaks, and ATR-CHK1, which responds to agents interfering with replication forks and single-strand breaks^48,49^. One of the earliest modifications of chromatin in the DNA damage response is phosphorylation of γH2AX, located at the sites of DNA strand breaks as immunoreactive foci. Expression of γH2AX was detected by both western blot analysis and confocal microscopy by 48 h in melanoma cells treated with **1** or **2**. It was previously reported that inhibition of HH signaling with GANT61 leads to alterations in genes involved in DNA damage. In particular, it was shown that GANT61 elicits a DNA damage response in colon cancer cells through the ATR/CHK1 axis^50^.

In this study, we show that **1** and, to a lesser extent, **2** overcome the G2 checkpoint despite large DNA damage, leading to the activation of the “mitotic death” program of mitotic catastrophe in response to replication stress (Fig. 7f). These results highlight a novel mechanism through which these SMO inhibitors might induce cell death in melanoma cells. Mitotic catastrophe is a tumor-suppressive mechanism, defined as a mode of cell death that results from aberrant mitosis^51^. Mitotic catastrophe can be induced by several drugs, including spindle assembly inhibitors, DNA-damaging agents and radiation^52^. It ensues from a combination of dysfunctional cell cycle checkpoints, particularly those related to DNA structure and spindle assembly, together with cellular damage^53^. At the moment, the molecular mechanisms that link mitotic catastrophe to the engagement of the apoptotic machinery in melanoma cells upon treatment with **1** or **2** are unknown and are under investigation. Nevertheless, mitotic catastrophe can be viewed as a desirable outcome for the development of novel anticancer drugs^31^. First, a considerable amount of cancer cells are tetraploid or aneuploid, rendering them more prone to mitotic aberrations and therefore particularly sensitive to the induction of mitotic catastrophe. Second, several chemotherapeutic drugs are currently used at concentrations that induce apoptosis irrespective of the cell cycle phase, yet are very efficient at triggering mitotic catastrophe at lower doses, which would significantly limit side effects.

In conclusion, we report the synthesis and the biological characterization of the most potent SMO inhibitors of the acylguanidine family discovered so far. Our findings highlight the enhanced HH pathway inhibition and anticancer properties of these compounds, opening the avenue for novel therapeutics for melanoma and, possibly, other types of cancer with active HH signaling.

## Materials and methods

### Cell lines and treatments

Commercial human melanoma cell lines A375 and MeWo and murine NIH3T3 cells were obtained from ATCC (Manassas, VA, USA). Cells were maintained in Dulbecco’s modified Eagle’s medium (DMEM) (Euroclone, Milan, Italy) supplemented with 10% fetal bovine serum (FBS), 1% penicillin-streptomycin, 1% glutamine (Lonza, Basel, Switzerland). Patient-derived SSM2c melanoma cells were previously described^54^, and were grown in DMEM/F12 (Euroclone) supplemented with 10% FBS, 1% penicillin-streptomycin, 1% glutamine (Lonza) and epidermal growth factor (EGF) (5ng/ml) (Life Technologies, Paisley, UK). Cells were periodically screened for mycoplasma contamination by PCR. For cell viability assay, 15,000 cells per well were plated in 12-well plates and treated with LDE-225 (Selleckchem, Munich, Germany). Compounds **1**, **2,** or **3** were used at the indicated concentrations for 48 h in 1% FBS. Nocodazole (Sigma-Aldrich, St Louis, MO, USA) was used at 600 nM for 16 h.

### Luciferase reporter assays

Luciferase reporters were used in combination with *Renilla* luciferase pRL-TK reporter vector (Promega, Madison, WI, USA) to normalize luciferase activities; pGL3Basic vector (Promega) was used to equal DNA amounts. Luminescence was measured using the Dual-Glo Luciferase Assay System (Promega) and the GloMax® 20/20 Luminometer (Promega).

### Lentiviral vectors and plasmids

Lentiviruses were produced in HEK-293T cells. Lentiviral vectors pLKO.1-puro and pLKO.1-shSMO were already described^40^. Cells were transfected with equal amounts of pCS2-Myc-tagged human GLI1 or pCS2-Myc.

### Quantitative real-time PCR

Total RNA was isolated with TriPure Isolation Reagent (Roche Diagnostics, Basel, Switzerland), subjected to DNase I treatment (Roche Diagnostics). Reverse transcription was performed with High Capacity cDNA Reverse Transcription Kit (Applied Biosystems, Carlsbad, CA, USA). Quantitative real-time PCR (qPCR) amplifications were carried out at 60 °C using FastStart SYBR Green Master (Roche Diagnostics) in a Rotorgene-Q. Primer sequences are: β-ACTIN fwd: 5ʹ-GAAAATCTGGCACCACACCT-3ʹ; β-ACTIN rev: 5ʹ-TAGCACAGCCTGGATAGCAA-3ʹ; GAPDH fwd: 5ʹ-GACGCTGGGGCTGGCATTG-3ʹ; GAPDH rev: 5ʹ-GCTGGTGGTCCAGGGGTC-3ʹ; TBP fwd: 5ʹ-CAACAGCCTGCCACCTTAC-3ʹ; TBP rev: 5ʹ-CTGAATAGGCTGTGGGGTC-3ʹ; GLI1 fwd: 5ʹ-CCCAGTACATGCTGGTGGTT-3ʹ; GLI1 rev: 5ʹ-GCTTTACTGCAGCCCTCGT-3ʹ; SMO fwd: 5ʹ-GGGAGGCTACTTCCTCATCC-3ʹ; SMO rev: 5ʹ-GGCAGCTGAAGGTAATGAGC-3ʹ.

### Primary sphere formation and self-renewal assay

For melanoma-sphere cultures, cells were seeded in human embryonic stem cell medium supplemented with 4 ng/ml basic fibroblast growth factor (bFGF), as previously reported^40,41,54^. For primary sphere formation assay, melanoma cells were seeded in 12-well plates at 1 cell/μl dilution and spheres were counted after 96 h. For self-renewal assay, primary melanoma spheres were dissociated into single cells and plated at 1 cell/μl dilution in 12-well plates. After 1 week, secondary spheres were counted.

### Flow cytometric analysis

For cell cycle analysis, melanoma cells were resuspended in 50 μg/ml propidium iodide, 0,1% Triton X-100 and 0.1% sodium citrate 24 h after treatment with compound **1**, **2** or vehicle (DMSO). Data were collected on BD Accuri C6 software and analyzed using ModFit LT software (Verity Software House). For the mitotic shake off procedure, cells were treated for 16 h with 600 nM of nocodazole. Typically, ≈95% cells were in mitosis after nocodazole removal. Cells were then washed with complete medium and treated with **1**, **2** or vehicle at indicated concentration in 1% FBS. Cell cycle distribution was analyzed every 2 h by using flow cytometry analysis. For apoptosis, melanoma cells were measured 48 h after treatment with **1** or **2** using Annexin V-PE/7-AAD apoptosis kit (BD Biosciences, San Jose, CA, USA), according to the manufacturer’s instructions. The number of both early (Annexin V^+^/7-AAD^−^) and late (Annexin V^+^/7-AAD^+^) apoptotic cells was detected and analyzed using BD Accuri C6 software.

### Western blot analysis

Western blotting was performed as already described^55^. The following antibodies were used: mouse anti-GLI1 (#2643), mouse anti-cyclin A2 (#4656), rabbit anti-BCL2 (#2876), rabbit anti-BAX (#2772), rabbit anti-cyclin B1 (#12231), rabbit anti-PARP-1 (#9532), rabbit anti-phospho-ATR (Ser428) (#2853), rabbit anti-phospho-CHK1 (Ser345) (#2348), rabbit anti-phospho-CDC2 (Tyr15) (#4539), rabbit anti-phospho-H2A.X (Ser139) (#9718), rabbit anti-phospho-Histone H3 (Ser10) (#3377), rabbit anti-phospho-WEE1 (Ser642) (#4910) (Cell Signaling Technology, Danvers, MA, USA), rabbit anti-CDC2 (sc-954), mouse anti-Myc (sc-40), mouse anti-HSP90 (sc-13119) (Santa Cruz Biotechnology, Santa Cruz, CA, USA), and rabbit anti-SMO (ST1718) (Merck Millipore, Burlington, MA, USA). Chemiluminescent detection was used. Cell fractionation was performed as previously described^56^. The following antibodies were used: mouse anti-GLI1 (#2643) (Cell Signaling Technology), goat anti-fibrillarin (D-14), and goat anti-GAPDH (V-18) (Santa Cruz Biotechnology).

### Immunofluorescence and confocal microscopy

For immunofluorescence experiments, A375 cells were seeded at a density of 5 × 10^3^ in 12-well cluster plates in DMEM supplemented with 1% FBS. After 24 h, cells were treated with compounds **1** or **2** (0.5 and 1 μM for 48 h). Cells were then washed with phosphate-buffered saline (PBS), fixed with ice-cold methanol for 5 min and permeabilized with Triton 0.2% in PBS for 10 min. Immunostaining of γH2AX was performed as previously described^57^. For Wheat Germ Agglutinin (WGA) immunostaining, cells were labeled as previously described^58^. Samples were visualized on a TSC SP5 confocal microscope (Leica Microsystems, Milan, Italy) installed on an inverted LEICA DMI 6000CS microscope, using PlanApo 40 × 1.25 NA objective or PlanApo 63 × 1.4 NA oil immersion objectives. Images were acquired using the LAS AF acquisition software (Leica Microsystems). Fluorescence intensity measurements were performed using the Quantitation Module of Volocity software (Perkin Elmer Life Science, Milan, Italy).

### Xenografts

A375 cells were resuspended in Matrigel (Becton Dickinson, Milan, Italy)/DMEM (1/1) and subcutaneously injected (10,000 cells per injection) into lateral flanks of adult (8 weeks) female athymic nude mice (Foxn1 nu/nu) (Envigo, Udine, Italy), as previously described^55,56^. Once tumors were palpable, mice were randomized in two groups and treated i.p. twice a day with comp **1** (15 mg/kg) dissolved in vehicle (30% 2-hydroxypropyl-β-cyclodextrin) (Sigma-Aldrich) or vehicle alone for 12 days. Subcutaneous tumor size was measured three times a week with a caliper and tumor volumes were calculated using the formula: V = W^2^ × L × 0.5, where W and L are, respectively, tumor width and length. The experiments were approved by the Italian Ministry of Health and were in accordance with the Italian guidelines and regulations.

### Statistical analysis

Data represent mean ± SD or mean ± SEM values calculated on at least three independent experiments. The *p*-values were calculated using one-way analysis of variance or Student’s *t*-test. A two-tailed value of *p* < 0.05 was considered statistically significant.

## Electronic supplementary material

Supplementary Information(DOCX 52 kb)

Supplementary Figure 1(EPS 3066 kb)

Supplementary Figure 2(EPS 2044 kb)

Supplementary Figure 3(EPS 2249 kb)

Supplementary Figure 4(EPS 1763 kb)

Supplementary Figure 5(EPS 6110 kb)

Supplementary Figure 6(EPS 2007 kb)

Supplementary Figure 7(EPS 5702 kb)

Supplementary Figure 8(EPS 5179 kb)

Supplementary Figure 9(EPS 4923 kb)

Supplementary Figure 10(EPS 1554 kb)
