# Supplementary material for: Targeted inhibition of Hedgehog-GLI signaling by novel acylguanidine derivatives inhibits melanoma cell growth by inducing replication stress and mitotic catastrophe
Source: Cell Death Dis. 2018 Feb 2;9(2):142. doi: 10.1038/s41419-017-0142-0 (PMC5833413; doi:10.1038/s41419-017-0142-0)
Supplement: Supplementary file 1 — Supplementary Information(DOCX 52 kb) [file 41419_2017_142_MOESM1_ESM.docx]

**Supplementary Information**

**Targeted inhibition of Hedgehog-GLI signaling by novel acylguanidine derivatives inhibits melanoma cell growth by inducing replication stress and mitotic catastrophe**

Silvia Pietrobono^1§^, Roberta Santini^1§^, Sinforosa Gagliardi^1^, Francesca Dapporto^2^, David Colecchia^2^, Mario Chiariello^2^, Cosima Leone^3^, Massimo Valoti^3^, Fabrizio Manetti^4^, Elena Petricci^4^, Maurizio Taddei^4^, Barbara Stecca^1,5^

**Supplementary Methods**

**Kinase assay**

The kinase assay was performed as described in Kinase Profile Service (Cerep, France, [www.cerep.fr](http://www.cerep.fr)).

**Synthesis of compounds 1-3.**

All reagents were used as purchased from commercial suppliers (Sigma Aldrich, St Louis, MI, USA; AlfaAesar, Haverhill, MA, USA) without further purification. The reactions were carried out in oven dried or flamed vessels. Solvents were dried and purified by conventional methods prior use. Toluene was distilled from sodium using benzophenone as the dryness detector. Flash column chromatography was performed with Merck silica gel 60, 0.040-0.063 mm (230-400 mesh). Aldrich or Merck aluminium backed plates pre-coated with silica gel 60 (UV254) were used for analytical and preparative thin layer chromatography and were visualized by staining with a ninhydrine or KMnO_4_ solution. NMR spectra were recorded at 25°C and 400 MHz for ^1^H and 100 MHz for ^13^C. The solvent is specified for each spectrum. Splitting patterns are designated as s, singlet; d, doublet; t, triplet; q, quartet; m, multiplet; br, broad. Chemical shifts (δ) are given in ppm relative to the resonance of their respective residual solvent peak. Mass spectroscopy analyses were recorded by electrospray ionization. The purity of all the compounds tested was checked by HPLC/MS analysis (as reported in Supplementary Information) resulting always higher than 95%.

General procedure for the synthesis of **1-2**: To a MeOH (2 ml) solution of **10** (42 mg, 0.12 mmol) a 10% HCl in MeOH solution (1.5 ml) was added. The mixture was stirred at r.t. under N_2_ overnight. The precipitate formed during the night was filtered and washed with PE (2 x 10 ml) and the solid obtained directly suspended in toluene (20 ml) in the presence of *N*-cyano-3,4,5-trimethoxybenzamide (26 mg, 0.11 mmol). The reaction mixture was stirred at reflux overnight. The solvent was removed under vacuum and the solid obtained was dissolved in MeOH (2 ml), a 10% HCl in MeOH solution (1.5 ml) was added. The mixture was stirred at r.t. under N_2_ overnight.

*3,4,5-Trimethoxy-N-(N-(4-methyl-3-(4-phenethylbenzamido)phenyl)carbamimidoyl) benzamide hydrochloride (****1****):* ^1^H NMR (400 MHz, Methanol-*d*_4_) δ 12.47 (bs, 1H), 11.55 (bs, 1H), 9.90 (bs, 1H), 7.96 (s, 1H), 7.75 (d, *J* = 8.0 Hz, 2H), 7.67 (s, 1H), 7.24 (s, 1H), 7.24 (t, *J* = 7.6 Hz, 2H), 7.17 (d, *J* = 6.8 Hz, 2H), 7.12 (d, *J* = 8.0 Hz, 1H), 3.82 (s, 9H), 2.96-2.90 (t, *J* = 2.0 Hz, 4H), 2.22 (s, 3H). ^13^C NMR (101 MHz, Methanol-*d*_4_) δ 176.35, 165.47, 159.01, 146.00, 140.59, 140.31, 136.35, 134.35, 134.78, 132.85, 131.51, 131.23, 131.23, 128.56, 126.01, 127.48, 126.84, 125.71, 120.62, 118.85, 105.54, 60.41, 55.53, 37.21, 36.98, 16.94. ES-MS: 567 [M + H]^+^, 589 [M + Na]^+^. Chemical formula C_33_H_35_ClN_4_O_5_; elemental analysis (H, C, N) calcd. C, 65.72; H, 5.85; Cl, 5.88; N, 9.29; O, 13.26; found C, 65.75; H, 5.82; N, 9.31.

*N-(N-(3-(4-(2-fluorophenethyl)benzamido)-4-methylphenyl)carbamimidoyl)-3,4,5-trimethoxybenzamide hydrochloride (****2****):* ^1^H NMR (400 MHz, Methanol-*d*_4_) δ 7.90-6.87 (m, 13H); 3.83 (s, 9H); 2.94 (s, 4H); 2.24 (s, 3H). ES-MS: 585 [M + H]^+^. ^13^C NMR (101 MHz, Methanol-*d*_4_) δ 167.51, 165.55, 161.12, 158.73, 146.00, 140.59, 140.31, 136.35, 134.35, 134.78, 132.85, 132.10, 131.51, 131.23, 131.23, 126.84, 125.71, 120.62, 118.85, 115.56, 105.54, 60.41, 55.53, 37.19, 36.85, 16.94. Chromatography: AcOEt/PE (3:7). Yield 43%. Chemical formula: C_33_H_34_ClFN_4_O_5_; elemental analysis (H, C, N) calcd. C, 63.82; H, 5.52; Cl, 5.71; F, 3.06; N, 9.02; O, 12.88; found C, 63.79; H, 5.50; N, 9.07.

*Synthesis of 3,4,5-trimethoxy-N-((4-methyl-3-(4-phenethylbenzamido)phenyl)carbamothioyl) benzamide (****3****)*: 3,4,5-trimethoxybenzoyl chloride (21 mg, 0.09 mmol, 1 eq.) was dissolved in dry acetone (20 ml) and NH_4_SCN (8.2 mg, 0.11 mmol, 1.2 eq.) was added. The suspension obtained was stirred at reflux for 2h and **10a** (30 mg, 0.09 mmol, 1 eq.) was added. The reaction mixture was refluxed for additional 3h under N_2_ and directly poured into a backer previously fill with ice. The solid formed was filtered in a büchner funnel and washed with H_2_O (2 x 20 ml), and PE (2 x 20 ml). The solid was purified by flash chromatography (AcOEt/PE: 4:1) obtaining a pale yellow solid in 20% yield.

^1^H NMR: (400 MHz, Methanol-*d*_4_) δ 12.52 (s, 1H); 8.98 (s, 1H); 8.38 (s, 1H); 7.79-7.46 (m, 14H); 3.93 (s, 9H); 2.99-2.94 (m, 4H); 2.33 (s, 1H). ^13^C NMR (101 MHz, Methanol-*d*_4_) δ 180.32, 165.47, 159.01, 146.00, 140.59, 140.31, 136.35, 134.35, 134.78, 132.85, 131.51, 131.23, 131.23, 128.56, 126.01, 127.48, 126.84, 125.71, 120.62, 118.85, 105.54, 60.41, 55.53, 37.21, 36.98, 16.94. ES-MS: 606 [M + Na]^+^. Chemical Formula: C_33_H_33_N_3_O_5_S elemental analysis (H, C, N) calcd. C, 67.90; H, 5.70; N, 7.20; O, 13.70; S, 5.49; found C, 67.93; H, 5.68; N, 7.22.

**Synthesis of compounds 7-10**

*General procedure for the synthesis of (E)-4-styrylbenzoic acid* derivatives ***7* (a-b)**: 4-((bromotriphenyl-5-phosphanyl)methyl)benzoic acid **5** (2.04 mmol, 1 eq.) was dissolved in dry THF (36 ml) and the reaction mixture was cooled down to -78°C. A 2.5 M solution of BuLi (3.4 ml, 3.5 eq.) in hexane was added and stirred at -78°C for 1h under N_2_. The proper benzaldehyde (3.06 mmol, 1.5 eq.) was added at 0°C and the orange solution obtained was stirred at r.t. overnight. AcOEt (60 ml) was added and washed with H_2_O (3 x 20 ml). 1N HCl was added to the water phase until pH 2 and extract with AcOEt (3 x 20 ml). The organic phases were washed with Brine (2 x 20 ml), dryed over dry Na_2_SO_4_ and evaporated under reduced pressure after filtration. The crude obtained was purified by flash chromatography.

*(E)-4-Styrylbenzoic acid (****7a****):* ^1^H NMR (400 MHz, DMSO-*d*_6_) 7.96 (d, *J* = 8 Hz, 2H), 7.72 (d, *J* = 8 Hz, 2H), 7.64 (d, *J* = 7.6 Hz, 2H), 7.45-7.26 (m, 11H). 223 [M - H]^-^. Chromatography: CHCl_3_/CH_3_OH (9:1). Yield 70%.

*(E)-4-(2-Fluorostyryl)benzoic acid (****7b****):* ^1^H NMR (400 MHz, Methanol-*d*_4_) ä 7.98 (d, *J* = 8.1 Hz, 2H), 7.82 (d, *J* = 8.2 Hz, 1H), 7.69 (t, *J* = 8 Hz, 1H), 7.63 (d, *J* = 8.1 Hz, 2H), 7.31-6.94 (m, 3H), 6.76 (d, *J* = 12 Hz, 1H) ;6.68 (d, *J* = 12 Hz, 1H). ES-MS: 241 [M - H]^-^. Chromatography: AcOEt/PE (1:3). Yield 40%.

*General procedure for the synthesis of 4-phenethylbenzoic acid* derivatives **8 (a-b)**: to a MeOH (10 ml) solution of **7** (1.86 mmol, 1 eq.), Pd/C 5% (0.09 mmol, 0.05 eq.) was added under N_2_. After 3 cycles N_2_/vacuum, the reaction mixture was stirred at r.t. under H_2_ overnight. The suspension was filtered over a celite pad and washed with MeOH (3 x 20 ml). The solvent was removed under reduced pressure and purified by flash chromatography.

*4-Phenethylbenzoic acid (****8a****):* ^1^H NMR (400 MHz, CDCl_3_) δ 8.01 (d, *J* = 8 Hz, 2H); 7.28-7.13 (m, 7H); 3.0-2.9 (m, 4H). ES-MS: 225 [M - H]^-^. Chromatography: AcOEt/PE (1:1). Yield 75%.

*4-(2-Fluorophenethyl)benzoic acid (****8b****):* (400 MHz, Methanol-*d*_4_) δ 7.88-7.85 (m, 2H); 7.25-6.89 (m, 6H); 2.92 (s, 4H). ES-MS: 243 [M - H]^-^. Chromatography: AcOEt/PE (1:1). Yield 71%.

*General procedure for the synthesis of* ***9 (a-b)***: **8** (1.1 mmol, 1 eq.) was dissolved in SOCl_2_

(10 ml) and the mixture was refluxed overnight. The solvent was evaporated and the product obtained was dissolved in dry CH_2_Cl_2_ (15 ml) and the solution was cooled down to 0°C. 2-Methyl-5-nitro aniline **4** (0.93 mmol, 0.8 eq.) and pyridine (2.24 mmol, 2 eq.) were added. After 10 min., the reaction mixture was heated up to r.t. and stirred overnight under N_2_. After dilution with CH_2_Cl_2_ (20 ml), the reaction mixture was washed with 1N HCl (3 x 15 ml), a saturated solution of NaHCO_3_ (3 x 15 ml), and Brine (2 x 15 ml). The organic phase was dryed over dry Na_2_SO_4_ and, after filtration and evaporation under reduced pressure, the crude was purified by flash chromatography.

*N-(2-Methyl-5-nitrophenyl)-4-phenethylbenzamide (****9a****):* ^1^H NMR (400 MHz, CDCl_3_) δ 8.92 (s, 1H); 7.94 (d, *J* = 8 Hz, 1H); 7.78 (d, *J* = 4 Hz, 2H); 7.72 (s, 1H); 7.35 (d, *J* = 8 Hz, 1H); 7.29-7.13 (m, 7H); 3.02-2.92 (m, 4H); 2.45 (s, 3H). ES-MS: 361 [M + H]^+^, 383 [M + Na]^+^. Chromatography: AcOEt/PE (1:4). Yield 56%.

*4-(2-Fluorophenethyl)-N-(2-methyl-5-nitrophenyl)benzamide* *(****9b****):* ^1^H NMR (400 MHz, CDCl_3_) δ 8.87 (s, 1H); 7.98-7.91 (m, 2H); 7.78 (d, *J* = 8.4 Hz, 2H); 7.35-6.98 (m, 7H); 2.97 (s, 4H); 2.41 (s, 3H). ^13^C NMR (101 MHz, CDCl_3_) δ 165.10, 146.49, 146.06, 136.28, 135.83, 131.32, 130.54, 130.54, 130.25, 129.86, 128.70, 127.50, 126.79, 123.56, 119.31, 117.43, 114.97, 114.75, 35.82, 30.36, 17.70. ES-MS: 401 [M + Na]^+^, 377 [M - H]^-^. Chromatography: AcOEt/PE (1:1). Yield 58%.

*General procedure for the synthesis of* ***10 (a-b)***: to a solution of **9** (0.34 mmol, 1 eq.) in MeOH (30 ml), Pd/C 5% (0.017 mmol, 0.05 eq.) was added under N_2_. After 3 cycles N_2_/vacuum, the reaction mixture was stirred at r.t. under H_2_ overnight. The suspension was filtered over a celite pad and washed with MeOH (3 x 20 ml). The solvent was removed under reduced pressure and purified by flash chromatography.

*N-(5-Amino-2-methylphenyl)-4-phenethylbenzamide (****10a****):*  ^1^H NMR (400 MHz, CDCl_3_) δ 7.76 (d, *J* = 12 Hz, 2H); 7.69 (s, 1H); 7.47 (s, 1H); 7.28-7.15 (m, 6H); 6.95 (d, *J* = 8 Hz, 1H); 6.42 (d, *J* = 8 Hz, 1H); 3.45 (s, 2H); 3.01-2.91 (m, 4H); 2.19 (s, 1H). ES-MS: 332 [M + H]^+^. Chromatography: AcOEt/PE (1:1).Yield 65%.

*N-(5-Amino-2-methylphenyl)-4-(2-fluorophenethyl)benzamide (****10b****):* ^1^H NMR (400 MHz, CDCl_3_) δ 7.77-6.93 (m, 10H); 6.44 (d, *J* = 8 Hz, 1H); 3.59 (s, 1H); 2.95 (s, 4H); 2.19 (s, 3H). Chromatography: AcOEt/PE (1:1). ES-MS: 349 [M + H]^+^. Yield 35%.

**Analysis of *in vitro* metabolic stability of 1 in rat (RLM) and human liver microsomes (HLM)**

Compound **1**, dissolved in MeCN/DMSO 3:1, was incubated separately at 1 μM (final concentration in 100 mM phosphate buffer, pH 7.4) with either 0.4 mg RLM or 0.3 mg HLM protein in a final volume 500 μl. The enzymatic reactions were initiated by addition of a NADPH-regenerating system (NADPH-GS) consisting of 2 mM β-NADPH, 10 mM glucose-6-phosphate, 0.4 U/ml glucose-6-phosphate dehydrogenase. Reactions were terminated at regular time intervals (overall range 0-30 min) by adding a double volume of cold MeCN. All incubations were performed in triplicate. HPLC analysis was performed on Agilent 1100 Series liquid chromatography system (Agilent Technologies, Palo Alto, CA, USA) equipped with a EC 150/4.6 nucleosil 100-3 C18 (Macherey-Nagel, Duren, Germany) and coupling with UV-VIS detector, setting at λ = 254 nm. Analysis was carried out using gradient elution of a binary solution; eluent A was MeCN with 0.1% HCOOH, while eluent B was an aqueous solution of HCOOH (0.1%). The analysis started at 20% A that rapidly increased up to 90% in 15 min and finally remaining at 90% A until 30 min. The analysis was performed at flow rate of 0.8 ml/min and injection volume was 40 µl. The intrinsic clearance (CL_int_) was calculated by the equation: CL_int_ = k[V]/[P] where k is the rate constant for the depletion of substrate (expressed in min^-1^), V is the volume of incubation in μl and P is the amount of microsomal proteins. For identification of the CYP-dependent metabolites, 10 μM and 30 μM **1** were incubated in presence of HLM or cDNA-expressed (supersomes) human CYP1A2, 2A6, 2C9 and 3A4 isoforms and NADPH-GS, as previously reported.^1^ After 60 min incubation reactions were stopped by adding MeCN. The samples were analysed by means Agilent HPLC/MS 1100 Series (MSD) operating in electrospray ionization (ESI) at 70 eV in positive manner, using the same chromatographic conditions reported above.

**Lentiviral vectors**

Lentiviruses were produced in HEK-293T cells. Lentiviral vectors LV-c and LV-shp53 were already described^2,3^.

**Supplementary RESULTS**

**Synthesis of compounds 1, 2 and 3.**

4-(Bromomethyl)benzoic acid **6** was treated with PPh_3_ in acetone at reflux for 6h affording **5** in quantitative yield (Supplementary Figure S1). A straightforward strategy involving a Wittig reaction between phosphonium salt **5** and benzaldehyde or 2-fluoro benzaldehyde with BuLi led to the expected benzoic acids **7 (a-b)**. Benzaldehyde showed a better reactivity than its 2-fluoro derivative, probably because of the steric hindrance of the fluorine in *orto* position. Double bond reduction with H_2_ and Pd/C led to **8 (a-b)** in good yields. The carboxylic acids were activated with SOCl_2_ and directly coupled with nitroaniline **4** obtaining, after reduction with H_2_ and Pd/C, **10(a-b)** in excellent yields. Aniline **10** was transformed into its hydrochloric acid salt and refluxed with *N*-cyano-3,4,5-trimethoxybenzamide in toluene. The acylguanidines obtained were treated with HCl in MeOH affording **1** and **2** in almost quantitative yields. Moreover, aniline **10a** was refluxed in acetone for 3h in the presence of 3,4,5-trimethoxybenzoyl chloride and NH_4_SCN to yield **3**. Compounds **1-3** were obtained as pure crystalline products and were directly used for biological assays. No degradation of **1** has been observed by HPLC at pH 7.4 (plasma), 10.4 and 4 even after 72h of incubation at 37°C.

***In vitro* metabolic stability of 1 in rat and human liver microsomes.**

Metabolic stability of **1** was assessed through incubation in rat (RLM) and human liver microsomes (HLM). Percent of the non-metabolized compound versus time were linearly related (Supplementary Figure S10a,b), suggesting a monoexponential relationship of the substrate depletion. As expected^1^ kinetic parameters (Supplementary Figure S10c) showed higher CL_int_ values in HLM than RLM. After incubation of **1** (1h) with HLM or RML, two new peaks (m/z of 553 and 583 amu) corresponding to *O*-demethylated and hydroxylated metabolites, were detected by HPLC/MS at higher retention time than the starting material. Only CYP2C9 and CYP3A4 were responsible for this metabolic pathway. These findings clearly indicated a significant stability of **1** either in RLM and HLM, as also resulted by the calculated intrinsic clearance CL_int_.

**Supplementary Figure LEGENDs**

**Supplementary Figure S1.** Schematic representation of synthesis of compounds **1**, **2** and **3**. *i.* PPh_3_, acetone, reflux, 6h; *ii. n*-BuLi, ArCHO, THF, from -78°C to r.t., 12h; *iii.* H_2_, Pd/C, MeOH; *iv.* SOCl_2_, r.t., 12h; *v.* **4**, Py, CH_2_Cl_2_, r.t., 12h; *vi.* H_2_, Pd/C, AcOH, MeOH; *vii.* HCl, MeOH, r.t., 12h; *viii.* *N*-cyano-3,4,5-trimethoxybenzamide, toluene, reflux, 3 h; *ix.* 3,4,5-trimethoxybenzoyl chloride, NH_4_SCN, acetone, reflux, 3h.

**Supplementary Figure S2.** Graphs showing kinase inhibition assay after incubation with 10 μM compound **1**. The y-axis (horizontal) depicts the mean activity (%) of two independent measurements (0.1% DMSO control was set to 100%). Hit thresholds are indicated by grey lines (80% activity). Data are shown are mean ± SD. No substantial inhibition against any of the enzymes was detected in presence of **1**.

**Supplementary Figure S3**. Determination of cell cycle analysis (**a-c**) and cell death by Annexin V/7AAD staining (**d-f**) in A375, SSM2c and Mewo cells treated with DMSO (0) or increasing doses of the SMO inhibitor LDE-225 for 72h. Data are shown as mean ± SD of at least three independent experiments. *, p<0.05 compared to DMSO control.

**Supplementary Figure S4**. (**a**) Western blot analysis of DNA damage markers pATM and pCHK2 in SSM2c and A375 cells treated with DMSO (0) or increasing doses of **1** for 48h. Cisplatin 10μM was used as control for DNA damage induction. ACTIN was used as loading control.

**Supplementary Figure S5**. (**a**) Western blot analysis of DNA damage markers in MeWo cells treated with DMSO (0) or increasing doses of **1** or **2** for 48h. HSP90 was used as loading control. (**b, c**) Evaluation of cell death by Annexin V/7AAD staining in MeWo cells treated with DMSO (0) or increasing doses of **1** (**b**) or **2** (**c**). (**d**) Western blot analysis of apoptotic markers in MeWo cells treated with DMSO (0) or increasing doses of **1** or **2** for 48h. HSP90 was used as a loading control. (**e, f**) Cell cycle analysis in MeWo cells treated with DMSO (0) or increasing doses of **1** or **2**. (**g**) Western blot analysis of cell cycle markers in MeWo cells treated with DMSO (0) or increasing doses of compound **1** or **2** for 24h. ACTIN was used as loading control. Data are shown as mean ± SD of at least three independent experiments. *, p<0.05 compared to DMSO control.

**Supplementary Figure S6.** Compound **1** induces G2/M cell cycle arrest in SSM2c cells. (**a**) Schematic representation of the protocol used to synchronize cells. (**b**) Effect of nocodazole treatment (660nM) for 16h. (**c**) Cells were released from nocodazole block and treated with DMSO or **1** (0.5 and 1μM) and cell cycle distribution was determined by flow cytometric analysis of propidium iodide stained cells collected at the indicated time points (right). Treatment with **1** prevents cell cycle progression after the released from nocodazole block.

**Supplementary Figure S7.** Effect of **2** on cell cycle distribution. **a**) Schematic representation of the protocol used to synchronize cells. (**b, c**) Effect of nocodazole treatment (660nM) in SSM2c (**b**) and A375 cells (**c**) for 16h. (**d, e**) SSM2c and A375 cells were released from nocodazole block and treated with DMSO or **2** (0.5 and 1μM) and cell cycle distribution was determined by flow cytometric analysis of propidium iodide stained cells collected at the indicated time points (right). (**f**) Western blot analysis of cell cycle markers in SSM2c and A375 cells treated with DMSO (0) or increasing doses of compound **2** for 24h. HSP90 was used as loading control.

**Supplementary Figure S8.** (a) Western blot analysis of p53, p53-Ser15 and γH2A.X in A375 and SSM2c cells transduced with LV-c or LV-shp53. ACTIN was used as loading control. (**b, c**) Evaluation of cell death by Annexin V/7AAD staining in A375 and SSM2c cells treated with DMSO (0) or increasing doses of **1** (**b**) or **2** (**c**). (**d**) Cell cycle analysis in A375 and SSM2c cells treated with DMSO (0) or increasing doses of **1** or **2**. *, p<0.05 compared to DMSO control.

**Supplementary Figure S9.** (**a**) Schematic representation of the protocol used to synchronize cells. (**b**) Effect of nocodazole treatment (660nM, 16h) in A375 cells transduced with LV-c or LV-shp53. Cells were released from nocodazole block and treated with DMSO or **1** (0.5 and 1μM) and cell cycle distribution was determined by flow cytometric analysis of propidium iodide stained cells collected at the indicated time points. (**c**) Western blot analysis of cell cycle markers in A375 cells transduced with LV-c or LV-shp53 and treated with DMSO (0) or increasing doses of compound **1** for 48h. ACTIN was used as loading control. (**d**) Western blot analysis of SSM2c cells transiently transfected with empty vector (pCS2) or GLI1 (pCS2-GLI1) and treated with **1** at the indicated doses for 48h. HSP90 was used as loading control.

**Supplementary Figure S10.** CYP-dependent metabolic stability of 1 µM compound **1** in rat (RLM) (**a**) and human (HLM) (**b**) liver microsomal preparations. Results are presented graphically in natural logarithm of percentage of compound recovery (100% at time 0 min) as a function of incubation time. Data are presented as mean ± SEM of three independent experiments. (**c**) Table shows *in vitro* metabolic stability of compound **1** in RLM and HLM liver microsomes.

**References**

1. D'Elia P, De Matteis F, Dragoni S, Shah A, Sgaragli G, Valoti M. DP7, a novel dihydropyridine multidrug resistance reverter, shows only weak inhibitory activity on human CYP3A enzyme(s). *Eur. J. Pharmacol.* 2009; **614**: 7-13.

2. Stecca B, Ruiz i Altaba A. A GLI1-p53 inhibitory loop controls neural stem cell and tumour cell numbers. *EMBO J.* 2009; 28: 663-676.

3. Pietrobono S, Morandi A, Gagliardi S, Gerlini G, Borgognoni L, Chiarugi P *et al.* Down-Regulation of SOX2 Underlies the Inhibitory Effects of the Triphenylmethane Gentian Violet on Melanoma Cell Self-Renewal and Survival. *J Invest. Dermatol* 2016; **136**: 2059-2069.
